# Supplementary material for: A Perilipin Affects Lipid Droplet Homeostasis and Aerial Hyphal Growth, but Has Only Small Effects on Virulence in the Insect Pathogenic Fungus Beauveria bassiana
Source: J Fungi (Basel). 2022 Jun 15;8(6):634. doi: 10.3390/jof8060634 (PMC9225014; doi:10.3390/jof8060634)
Supplement: Supplementary file 1 [file jof-08-00634-s001.zip › jof-1723234-supplementary.pdf]

Journal of *Fungi*

MDPI

Supplementary Materials

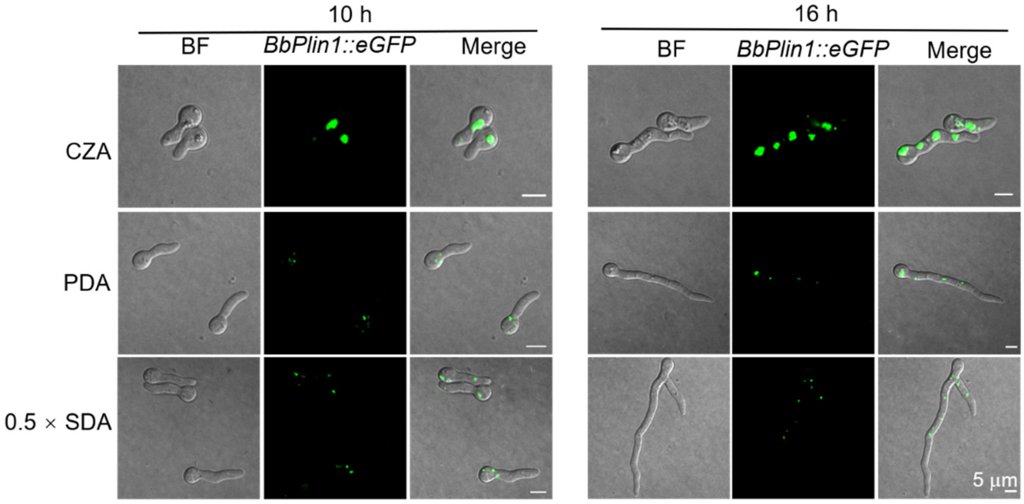

Figure S1. Protein expression analysis of BbPlin1::eGFP production on solid media. Fungal conidia were harvested from *BbPlin1::eGFP* grown on PDA for 2 weeks at 26°C. GFP signal was observed using the *B. bassiana BbPlin1::eGFP* strain cultured on CZA, PDA and 0.5 × SDA media for 10 and 18 h at 26°C. BF, bright field. Bar, 5 μm.

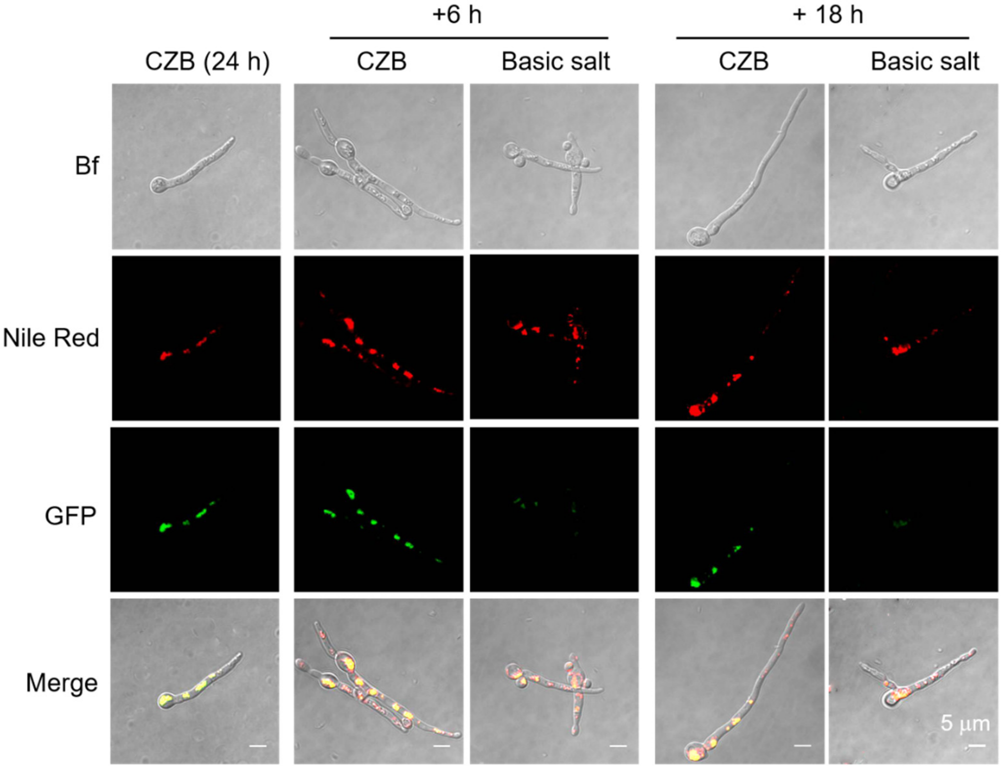

Figure S2. Protein expression analysis of BbPlin1::eGFP production under starvation conditions. Fungal conidia were harvested from *BbPlin1::eGFP* grown on PDA for 2 weeks at 26°C. The *B. bassiana BbPlin1::eGFP* strain was pre–cultured in CZB for 24 h and fungal cells were collected by centrifugation and washed with sterile water. Fungal cells were then inoculated into basic salts solution without nutrients and cultured for 6 and 18 h before visualization via confocal fluorescent microscopy. BF, bright field. Bar, 5 μm.

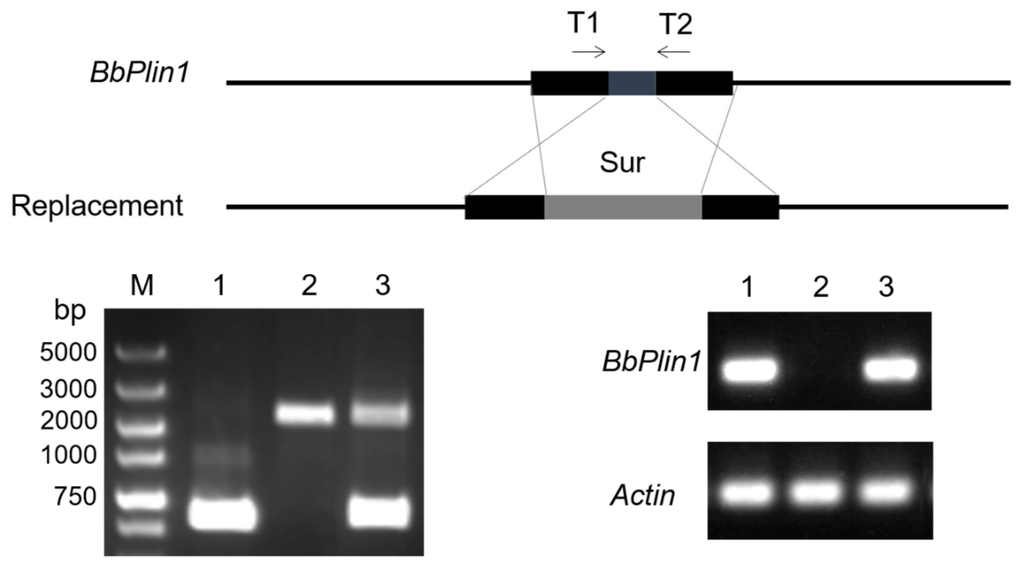

Figure S3. Screening of *B. bassiana* Δ*BbPlin1* knockout mutants. (A) Schematic of construction of *BbPlin1* mutants. (B) Confirmation of *BbPlin1* knockout strains by PCR. Lane M, Marker 5000. Lane 1–3 show the *B. bassiana* wild type, Δ*BbPlin1* mutant, and Δ*BbPLin1*^{C} complemented strains respectively. (C) Real–time PCR analysis of *BbPlin1* expression in indicated strains. All experiments were performed using three technical replicates and the entire experiment performed three times.

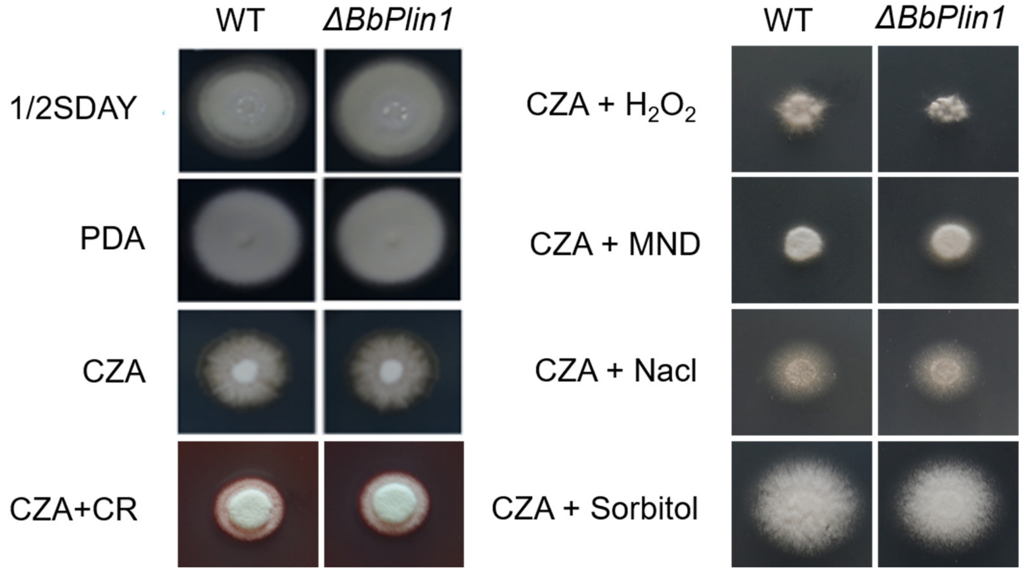

Figure S4. Effects of loss of *BbPlin1* on growth phenotypes and stress responses of *B. bassiana*. Fungal conidia were harvested from *B. bassiana* wild type (WT) and mutant (Δ*BbPlin1*) grown on PDA for 2 weeks at 26°C. Conidial suspensions (2 μl, 1 × 10^6 conidia/ml) of indicated fungal strains were inoculated on CZA, PDA, 0.5 × SDA, and CZA amended with indicated stress causing agents, including 3 mM H_2O_2, 30 mM MND, 0.7 M NaCl, 1.2 M Sorbitol, 25 μg/mL Congo Red (CR). Plates were incubated at 26°C for 8 d before being photographed.

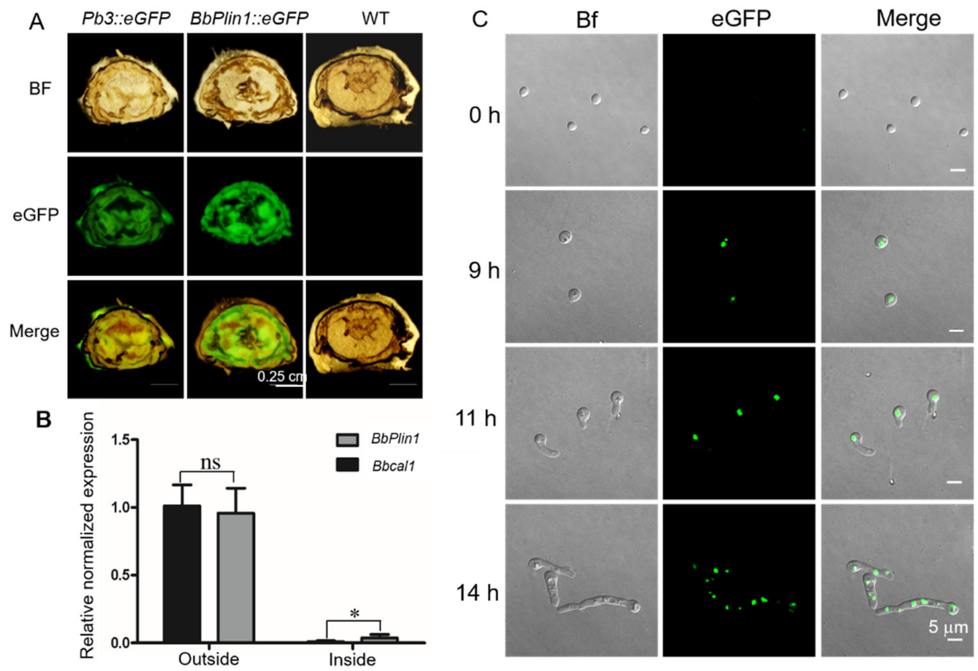

Figure S5. Expression analysis of *BbPlin1 B. bassiana* growing within versus growing on the surface of insect cadavers. (A) GFP signal of *B. bassiana* fungal cells harboring the *BbPlin1::eGFP* construct growing within the insect host and on the surface of cadavers observed in transverse sections of the cadavers. *B. bassiana* harboring the *Pb3::eGFP* construct which contains a constitutive promoter *Pgpda* (*Pb3*) druring eGFP expression was used as a positive control. (B) Expression analysis of *BbPlin1* and *BbCal1* in *B. bassiana* wild type cells isolated from growth (i) within, and (ii) on the surface of cadavers. (C) Expression analysis of *BbPlin1* of *B. bassiana PBbPlin1::eGFP* conidia harvest from cadavers and allowed to germinate in PDA media. Fungal conidia were harvested from indicated *B. bassiana* strains grown on PDA for 2 weeks at 26°C. BF, bright field. Bar, 5 μm. All experiments were performed using three technical replicates and the entire experiment performed three times. Stars represent statistical difference (* $p < 0.05$; ns, no significant, *t* test).

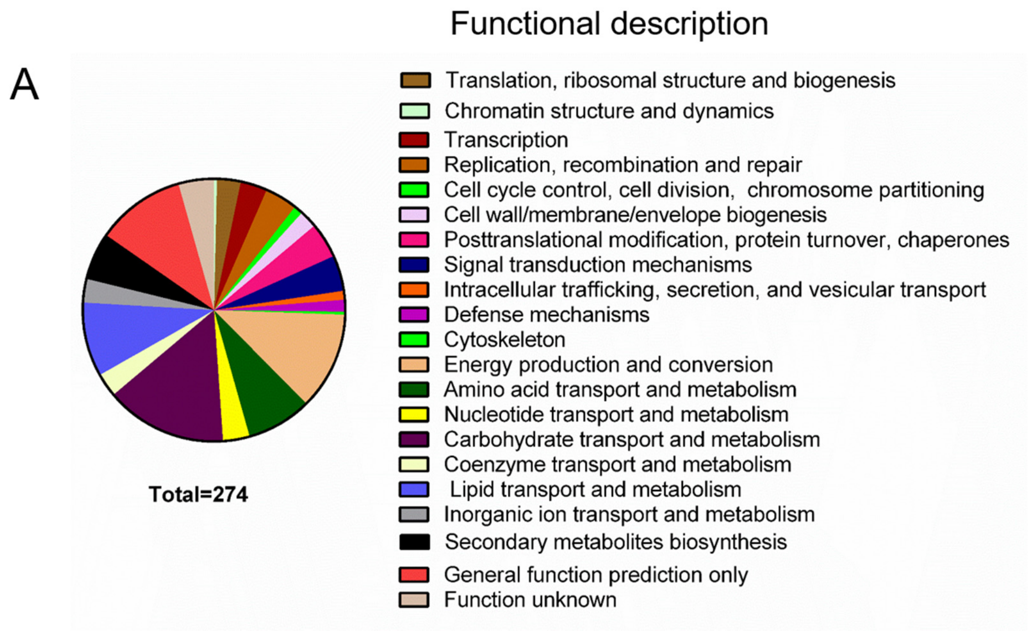

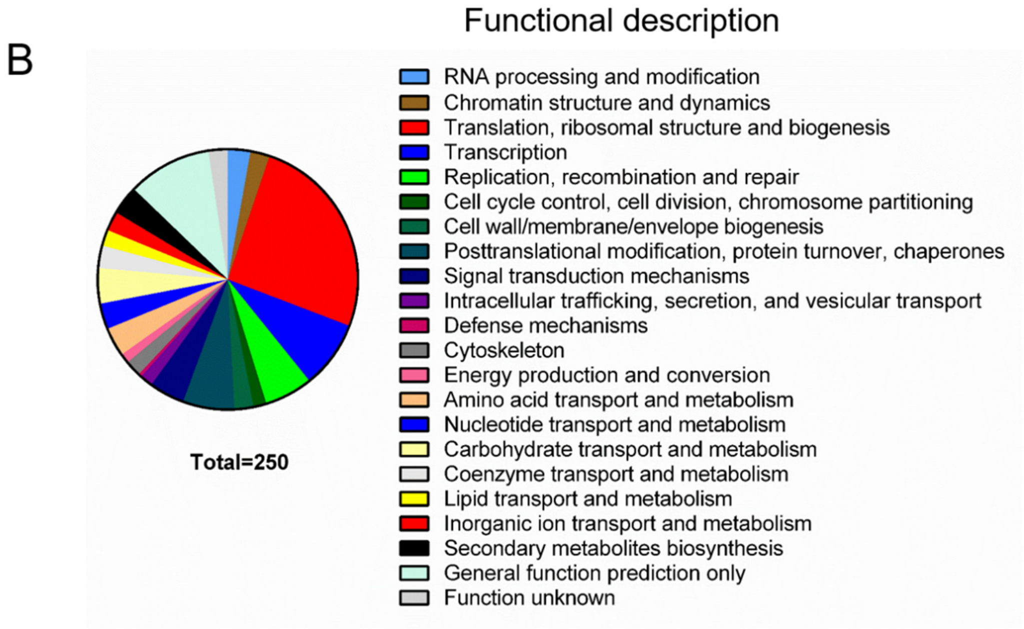

Figure S6. Overview of upregulated (A) and downregulated (B) genes in comparisons between Δ*BbPlin1* and *B. bassiana* wild type strains, and categorized according to putative functions gathered from Fungal Genome Database FunCat.

Table S1. Primers used in this study

Primer	Sequence (5'–3')	
P1–F	tatgaccatgattacgaattcaaattcgacgacagggtacg	Mutant construction LB
P1–R	tctgtcgacactagtgaattccgggtgcctgttattgttct	Mutant construction LB
P2–F	gaggtaatccttctttctagatgtcgagatatcccactcc	Mutant construction RB
P2–R	tgcctgcaggtcgactctagacatcatcccgacctttgact	Mutant construction RB
P3–F	gggaattctccttcattggcgcgttta	Fusion construction
P3–R	accagaaccaccctggttgattttctccttgac	Fusion construction
P4–F	tcaaccagggtggttctggtggtggttctggtatggtgagcaagggcgagga	eGFP
P4–R	agtctagatacttgtacagctcgtcca	eGFP
P5–F	ttgcacctatcgacgagtca//ttctccttgacctcctgggt	Mutant screening
P6–F	tgcgaggactgctatcaatg//atccaagtcatcgccatttc	Mutant screening
Bbcale–RT– F/–R	Gtccttatgacgacgacgcttc//ccgtgaggttgttcgtatga	Real–time PCR primers
BbPlin1–RT–F/–R	ctacttctccaagccctacc//actctgcgttgtagacttcg	Real–time PCR primers
Actin–RT–F/–R	ttggtgcgaaacttcagcgtctagtc//tccagcaaatgtggatctccaagcag	Actin primers
DGA1–RT–F/–R	aagcttttcgccggatattt//gcgaaagttggagtccagag	Real–time PCR primers
Tgl–RT–F/–R	ggctcgttgacaaaccaaat//gcgacaagtgaagcaatcaa	Real–time PCR primers
Enoyl–CoA–RT–F/–R	taatagcacgtttgaggcgc//ttgttcttccaggctcgtct	Real–time PCR primers

Table S2. The KEGG pathway analysis of DEGs involved in lipid metabolism

Pathway ID	Description	Gene number (ΔBbPlin1/WT_UP)	Gene number (ΔBbPlin1/WT_UP)
Map00071	Fatty acid degradation	2	1
Map00061	Fatty acid biosynthesis	1	
Map00062	Fatty acid elongation	1	1
map00564	Glycerophospholipid metabolism	3	1
map00561	Glycerolipid metabolism	7	0
map00563	Glycosylphosphatidylinositol (GPI)–anchor biosynthesis	2	0
map00600	Sphingolipid metabolism	1	4
map01040	Biosynthesis of unsaturated fatty acids	2	0
